# Neuroactive Steroid–Gut Microbiota Interaction in T2DM Diabetic Encephalopathy

**DOI:** 10.3390/biom13091325

**Published:** 2023-08-29

**Authors:** Silvia Diviccaro, Lucia Cioffi, Rocco Piazza, Donatella Caruso, Roberto Cosimo Melcangi, Silvia Giatti

**Affiliations:** 1 Dipartimento di Scienze Farmacologiche e Biomolecolari, Università degli Studi di Milano, 20133 Milan, Italy; silvia.diviccaro@unimi.it (S.D.); lucia.cioffi@unimi.it (L.C.); donatella.caruso@unimi.it (D.C.); roberto.melcangi@unimi.it (R.C.M.); 2 Dipartimento di Medicina e Chirurgia, Università di Milano—Bicocca, 20126 Milan, Italy; rocco.piazza@unimib.it

**Keywords:** memory, hippocampus, allopregnanolone, corticosterone, ZDF, *Collinsella*, *Paraprevotella*, *Phascolarctobacterium*, *Turicibacter*, *Romboutsia*

## Abstract

The pathological consequences of type 2 diabetes mellitus (T2DM) also involve the central nervous system; indeed, T2DM patients suffer from learning and memory disabilities with a higher risk of developing dementia. Although several factors have been proposed as possible contributors, how neuroactive steroids and the gut microbiome impact brain pathophysiology in T2DM remain unexplored. On this basis, in male Zucker diabetic fatty (ZDF) rats, we studied whether T2DM alters memory abilities using the novel object recognition test, neuroactive steroid levels by liquid chromatography–tandem mass spectrometry, hippocampal parameters using molecular assessments, and gut microbiome composition using 16S next-generation sequencing. Results obtained reveal that T2DM worsens memory abilities and that these are correlated with increased levels of corticosterone in plasma and with a decrease in allopregnanolone in the hippocampus, where neuroinflammation, oxidative stress, and mitochondrial dysfunction were reported. Interestingly, our analysis highlighted a small group of taxa strictly related to both memory impairment and neuroactive steroid levels. Overall, the data underline an interesting role for allopregnanolone and microbiota that may represent candidates for the development of therapeutic strategies.

## 1. Introduction

The prevalence of type 2 diabetes mellitus (T2DM) and its complications have increased in the past decades [1,2,3]. This is one of the most prevalent metabolic disorders worldwide, and obesity is a driving factor for developing T2DM. Major attention has focused on the consequences of the diabetic status on the kidneys, eyes, and peripheral nervous system; however, the brain is also affected. Indeed, considering the increased prevalence of DM and the decreasing age of its diagnosis, DM-related cognitive dysfunction will probably increase and have a substantial effect on society. Indeed, cognitive decline is increasingly recognized as an important comorbidity of diabetes mellitus [4,5]. Of relevance, several clinical trials have indicated that intensive peripheral glucose control has no significant effect on cognitive function, vascular complications, or total brain volume [6,7,8]. Moreover, cognitive impairment predisposes DM patients to treatment-related complications, such as severe hypo- or hyper-glycemic episodes [9,10]. In addition, patients with cognitive impairment have an increased risk of cardiovascular events and death compared with DM patients with intact cognition [10].

Brain abnormalities that characterize T2DM are learning and memory deficits as well as decreased information processing speed, attention, executive functions, and decreased brain volume [11,12,13,14]. Even though some factors have been identified, the exact mechanisms leading to diabetic encephalopathy remain elusive, and this acknowledges the lack of therapeutic intervention. The preclinical data indicate oxidative stress, mitochondrial damage, and inflammatory response as key players [15], but glucocorticoids also have a role. Glucocorticoids are the product of the hypothalamus–pituitary–adrenal gland (HPA) axis, and it has been reported that their levels are increased in T2DM patients [16,17,18]. Interestingly, it is well recognized that elevated glucocorticoid plasma levels are usually associated with declarative memory deficits, possibly through the action of glucocorticoids on sensitive structures, like the hippocampus [19,20]. Based on this premise, and accordingly with our previous experience in an experimental model of hyperglycemia [21,22], here we explored molecular targets possibly involved in diabetic encephalopathy in the well-established model of T2DM, the Zucker diabetic fatty (ZDF) rats.

Neuroactive steroids are defined as steroid molecules, like progestins, androgens, estrogens, and glucocorticoids, that are able to affect brain functions independently by their origin. They are involved in the physiological regulation of brain functions, including memory [23,24]. They are metabolized in the brain from steroids produced in the periphery or can be directly produced in the nervous system. Interestingly, alterations of neuroactive steroid levels have been reported in neurodegenerative conditions, and their rescue proved to be successful in many preclinical as well as some clinical studies due to their role as anti-inflammatory and antioxidant molecules [24,25,26,27,28]. Moreover, neuroactive steroids can improve memory and cognition, as recently reviewed [29]. Concerning diabetic encephalopathy, previous reports indicated a role for neuroactive steroids showing alteration in their levels in type 1 DM (T1DM) [21,30]; however, no observation in T2DM experimental models was reported.

Beyond neuroactive steroids, it is worth noting that brain functioning is the outcome of the brain–gut–microbiota communication since a microbiota-mediated modulation of host pathways exists [31]. Indeed, as suggested in germ-free mice, the absence of microbes affects the pool of neuroactive steroids in the brain, supporting evidence of the physiological role of the microbiome in the modulation of the steroid environment [32]. Interestingly, the lack of microbiome is related to the increase in corticosterone in plasma [33] and cognitive deficits [34], suggesting a role for microbes in the modulation of glucocorticoids, cognition, and memory. Moreover, in a model of T1DM, a significant correlation among gut microbiota, steroid molecules, and cognitive impairment was recently highlighted [35], underlining that specific taxa modifications are related to gut steroids and memory decline. Interestingly, recent data showed a possible link among cognitive decline, T2DM, and gut microbiota, where microbe modulation can improve behavioral impairment [36]. 

Overall, data in the literature indicate an interesting role for neuroactive steroids and microbiota in cognition and memory function, also concerning the diabetic condition. Indeed, even if our previous observation linking neuroactive steroids and microbiota was obtained in a model of T1DM, we were interested in evaluating whether these elements are altered in the ZDF rat, a different model of diabetes. Moreover, we also wondered whether neuroactive steroids and intestinal microbiota changes may be associated with memory impairment. On the other hand, evidence reporting information on these issues in T2DM is scarce or even absent. 

Thus, to fill this knowledge gap, here we report data supporting the interaction between neuroactive steroid levels and the microbiota composition observed in ZDF rats as compared to their lean controls. In particular, we studied memory impairment and molecular targets related to cognitive alterations in the brain area mainly involved in memory function, i.e., the hippocampus. Here, we evaluated oxidative stress, neuroinflammation, and mitochondrial parameters as well as levels of neuroactive steroids. In parallel, we analyzed glucocorticoid levels in plasma and the gut microbiome. Finally, we performed correlations among all these factors to retrieve some insights into the etiopathogenetic mechanism of diabetic encephalopathy and thus into some possible therapeutic agents.

## 2. Materials and Methods

### 2.1. Animals

Male Zucker diabetic fatty (ZDF, fa^−/−^ genotype) and lean (fa^+/−^ genotype) control rats (7-week-old at arrival, Charles Rivers Laboratories, Lecco, Italy) were used. Animals were housed in the animal care facility of the Dipartimento di Scienze Farmacologiche e Biomolecolari (Università degli Studi di Milano, Milan, Italy), in individually ventilated cages (IVC), with food and tap water available ad libitum. Standard housing conditions were the following: controlled temperature (21 ± 4 °C), humidity (40–60%), room ventilation (12.5 air changes per h), and light cycles (12 h light/dark cycle; on 7 A.M./off 7 P.M.). All animal procedures were performed in accordance with national (D.L. No. 26, 4 March 2014, G.U. No. 61 14 March 2014) and international laws and policies (EEC Council Directive 2010/63, 22 September 2010: Guide for the Care and Use of Laboratory Animals, United States National Research Council, 2011). The procedures were approved by the ethics committee of the Università degli Studi di Milano and by the Italian Ministry of Health (authorization number: 464/2021). 

### 2.2. Experimental Design

**Figure 1:**
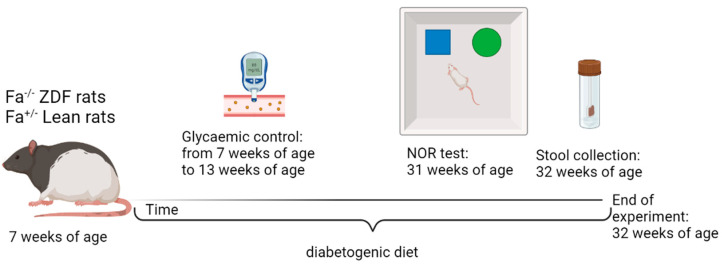
Flow chart scheme for experimental design. NOR test: novel object recognition test. Created with BioRender.com.

Starting from arrival, both lean controls and ZDF animals were fed with a diabetogenic diet (Purina 5008; Charles Rivers Laboratories, Lecco, Italy) until the end of the experiments, at 32 weeks of age. The glycemic condition was evaluated weekly starting from the arrival of the animals until 13 weeks of age by tail vein blood glucose measurement using a commercial glucometer (Contour next, Ascensia Diabetes Care Italy, Milan, Italy). At 11 weeks of age, all ZDF rats presented feeding blood glucose above 300 mg/dL. Body weight and animal well-being were assessed weekly and twice a week, respectively, throughout all the experiment. Memory function was evaluated at 31 weeks of age. At 32 weeks of age, all rats were euthanized under deep isoflurane anesthesia and the hippocampus and plasma were collected and stored at −80 °C until analyzed (Figure 1). Stool samples were collected 24 h before the sacrifice. Blood samples were collected in tubes with EDTA 0.25 M, and plasma was obtained by centrifugation at 2500× *g* for 15 min at 4 °C [37]. 

### 2.3. Novel Object Recognition (NOR) Test

At 31 weeks of age, animals were tested in a Plexiglas non-transparent open field, in an isolated room under low light conditions, in the absence of direct overhead lighting. The NOR test was performed as previously reported [22]. Briefly, animals were subjected to a 1 h adaptation session to the room; a 5 min session for the encoding phase; a 1 h retention phase; and 5 min of a testing session. In the adaptation phase, all the animals explored two identical objects for 5 min (trial session-encoding phase), completing a minimum of 15 s of time required for exploration. During the retention phase, rats were put back into their home cages. Then, the rats returned to the open field, where one of the objects presented previously was replaced by a novel one. During the encoding and testing sessions, the time spent by the animals in the exploration of the objects (i.e., sitting near the objects, sniffing, or touching them) was recorded by two independent observers, blind to the experimental design. The NOR index was calculated as follows: (exploration time of novel object/exploration time of novel + familiar object) × 100 [22].

### 2.4. Corticosterone ELISA Kit

For the evaluation of the corticosterone levels in plasma, an ELISA Kit (Catalog No. ADI−900−097; Enzo Life Sciences, Farmingdale, NY, USA) was used. Briefly, in this competitive enzyme immunoassay, the antigen competes for limited antibody binding sites with the antigen conjugated to a reporter enzyme, producing a negative relationship between substrate turnover and antigen concentration. Given the high concentration in plasma, all samples were diluted 1:25 times. Then, all steps reported by the protocol were followed as reported in [38]. The microplate reader read the optical density at 405 nm, with correction between 570 and 590 nm. 

### 2.5. Liquid Chromatography–Tandem Mass Spectrometry Analysis (LC–MS/MS) 

For the quantitative analysis of pregnenolone (PREG), progesterone (PROG), dihydroprogesterone (DHP), allopregnanolone (ALLO), isoallopregnanolone (ISOALLO), dehydroepiandrosterone (DHEA), testosterone (T), dihydrotestosterone (DHT), 5α-androstane−3α, 17β-diol (3α-diol), and 17β-Estradiol (17β-E), the hippocampus (60 mg) and plasma (300 μL) were extracted and purified as we previously described [37,39,40]. 17β-Estradiol−2,3,4−^13^C_3_ (^13^C_3_−17β-E) (2 ng/sample), progesterone−2,3,4,20,25−^13^C_5_ (^13^C_5_–PROG) (0.4 ng/sample), and pregnenolone−20,21−^13^C_2_−16,16 D_2_ (^13^C_2_D_2_–PREG) (10 ng/sample) were used as internal standards. Hippocampus samples were homogenized in 1% acetic acid in methanol (MeOH) using a Tissue Lyzer (Qiagen, Milan, Italy), and plasma samples were diluted in acetonitrile. After an overnight extraction at 4 °C, the organic phase was purified using organic phase extraction as previously described [37,39,40]. The quantitative analysis was performed using liquid chromatography (LC) supplied by Surveyor LC Pump Plus and Surveyor Autosampler Plus (Thermo Fisher Scientific, Waltham, MA, USA) connected with a linear ion trap-mass spectrometer LTQ (Thermo Fisher Scientific, Waltham, MA, USA), operated in positive atmospheric pressure chemical ionization (APCI+) mode. The chromatographic separation was achieved with a Hypersil Gold column C18 (100 mm × 2.1 mm, 3 μm; Thermo Fisher Scientific, Waltham, MA, USA) maintained at 40 °C. The mobile phases consisted of 0.1% formic acid in H_2_O (phase A) and 0.1% formic acid in MeOH (phase B). Gradient elution was as follows: 0–1.50 min 70% A; 1.50–2.00 min 55% A; 2.00–3.00 min 55% A; 3.00–35.00 min linear gradient to 36% A; 35.00–40.00 min 25% A; 41.00–45.00 min 1% A; 45.00–45.20 min 70% A; and 45.40–55.00 min equilibrate with 70% A. The 25 μL sample was injected at a flow rate of 250 μL/min. The divert valve was set at 0–8 min to waste, 8–45 min to source, and 45–55 min to waste. The injector needle was washed with MeOH/H_2_O 1:1 (*v*/*v*). LC–MS/MS data were acquired and processed using the software Excalibur^®^ release 2.0 SR2 (Thermo Fisher Scientific, Waltham, MA, USA). Quantitative analysis of neuroactive steroids was achieved based on calibration curves freshly prepared and extracted.

### 2.6. RNA and Protein Extraction

Total RNA and proteins from the hippocampus were extracted using Trizol (Invitrogen, San Giuliano Milanese, Italy). Briefly, tissues were homogenized with the Tissue Lyzer instrument (Qiagen, Milan, Italy), and chloroform was added to obtain phase separation. RNA was present in the upper aqueous phase, and its separation was obtained with a Directzol^TM^ RNA MiniPrep kit (Zymo Research, Irvine, CA, USA) in accordance with the manufacturer’s protocol and as previously reported [41].

Protein extraction was instead performed starting from the lower organic phase obtained by tissue homogenization and according to the Trizol reagent protocol, with minor modifications [42]. Briefly, after the separation of RNA and DNA from proteins, the phenolic-ethanol phase was combined with isopropanol, and the protein pellet was obtained by centrifugation (12,000× *g*, 10 min, 4 °C). This pellet was washed several times with 0.3 M guanidine hydrochloride in 95% ethanol and with 100% ethanol, and then air-dried. The protein pellet was mechanically resuspended with a needle in a solution *v*/*v* of SDS1% + UREA 8M, and incubated in a water bath at 50 °C for 40 min. The insoluble debris were pelleted through centrifugation at 10,000× *g*, 10 min, 4 °C, while the supernatant containing the proteins was quantified with BCA assay following the manufacturer’s protocol, and aliquots containing equal amounts of protein were prepared.

### 2.7. Real-Time Polymerase Chain Reaction

After RNA quantification by NanoDrop™2000 (ThermoFisher scientific, Milano, Italy), gene expression was evaluated by TaqMan quantitative real-time PCR using a CFX96 real-time system (Bio-Rad Laboratories, Segrate, Italy). Multiplexed reactions in 96-well format were prepared using the iTaq™ Universal Probes One-Step Kit (Bio-Rad, Segrate, Italy), as duplicate, including a normalizing internal control, 36B4 (Eurofins MWG Operon, Milano, Italy), and specific TaqMan MGB probes and primers purchased at Eurofins MWG Operon (Milano, Italy): IL−1β fwd: TGCAGGCTTCGAGATGAAC rev: GGGATTTTGTCGTTGCTTGTC; TNFα fwd: CTTCTCATTCCTGCTCGTGG rev: TGATCTGAGTGTGAGGGTCTG. Expression levels were obtained by interpolating a standard curved run with samples [43].

### 2.8. Western Blotting

To perform the Western blotting, previously prepared aliquots (see above) were heated to 100 °C or to 37 °C in case of OXPHOS, for 5 min, run on polyacrylamide gel, and transferred to nitrocellulose membranes. After cutting the membranes, blocking was performed with 10% non-fat dry milk or 5% bovine serum albumin (BSA) for 1 h at RT. Then, primary antibodies were incubated overnight at 4 °C on the membranes corresponding to the target proteins. Specifically, mouse OXPHOS (AB10413; Abcam, Cambridge, UK) was used at 1:2000, rabbit MFN2 (9482S) and mouse DRP1 (14647S; both from Cell Signaling Technology, Beverly, MA, USA) at 1:1000 in 2.5% non-fat dry milk, rabbit SOD2 (HPA001814; Sigma-Aldrich, Merk Life Science SRL, Milano, Italy) and rabbit OPA1 (80471S; Cell Signaling Technology, Beverly, MA, USA) were used at 1:1000 dilution in 5% BSA while rabbit GAPDH (SC_25778; Santa Cruz Biotechnology, Inc., Dallas, TX, USA) was used at 1:10,000 dilution in 2.5% non-fat dry milk. After extensive washing, the membranes were incubated for 2 h RT with an anti-rabbit or anti-mouse horseradish peroxidase-conjugated secondary antibody, according to the primary antibody, as indicated. The ECL method was used to detect protein bands, and the signals were acquired with a ChemiDoc^TM^ XRS+ system (Bio-Rad, Segrate, Italy) and analyzed with Image Lab^TM^ software version 5.2.1 (Bio-Rad, Segrate, Italy). The mean control value within a single experiment was set to 100, and all the other values were expressed as a percentage. This experiment was performed in accordance with a previously published report [44].

### 2.9. Thiobarbituric Acid-Reactive Substance

The hippocampal thiobarbituric acid–reactive substances (TBARS) assay was performed as an indirect index of reactive oxygen species (ROS) production. Indeed, this method measures lipid peroxidation, which is positively correlated with the ROS amount. The assay was performed as published previously [45]. Briefly, 400 μL of lysis buffer (Tris-HCl 0.1 M, pH 7.4; EDTA 1.34 mM; glutathione 0.65 mM) was added to 10 μg of tissue for homogenization. Then, 100 μL of homogenate was incubated at 95 °C for 1 h with a solution containing 600 μL of phosphoric acid 1% and 200 μL of TBA 0.6%. After incubation, samples were cooled to RT and 1 mL of n-butanol was added. Then, the extraction of TBARS was obtained with centrifugation at 1200× *g* at 4 °C for 20 min to obtain the supernatant that was then collected and analyzed fluorometrically at an excitation wavelength of 532 nm and an emission wavelength of 553 nm. Quantification was performed using the standard curve prepared with malondialdehyde following similar conditions.

### 2.10. 16S Next-Generation Sequencing

16S rRNA analysis was performed using 16A-V4 as the target region [46]. 16S rRNA amplicon sequencing was performed using Illumina technology at the DNA sequencing facility of GalSeq Srl [47]. Following sequencing, raw fastq reads were inspected using FastQC [48]. When required, adaptor sequences were removed using Cutadapt [49]. The filtered fastq reads were processed using QIIME2 [50] v. 2020.6. Rooted phylogenetic trees were generated using the phylogeny align-to-tree-mafft-fasttree pipeline. Rarefaction curves were calculated using the following metrics: chao1, Simpson, goods_coverage, faith_pd, Shannon, and observed_otus and used to test for sample saturation. Alpha diversity was calculated using the evenness, Shannon, Faith, and observed features metrics with the Wilcoxon rank sum test; beta diversity was calculated using Bray–Curtis, unweighted and weighted Unifrac; beta statistical analyses were performed using Anosim and Permanova. Multiple tests were controlled using the Benjamini–Hochberg procedure; tests with q values < 0.1 were considered statistically significant. Feature tables were manually inspected to select the appropriate sampling depth.

Taxonomy was annotated using SILVA as the reference database [51] with a pre-trained naive Bayes classifier optimized for the V4 region. Differential abundance in the two groups was calculated using ANCOMBC [52]; plots were generated using STAMP [53].

### 2.11. Statistical Analysis

The Kolmogorov–Smirnov test was used to control the normal distribution of the data (excluding those from 16S next-generation sequencing experiments, see above). All data presented a Gaussian distribution; indeed, they were analyzed by unpaired two-tailed Student’s *t*-test and considered significant when the *p*-value was < 0.05. For those data sets where the F test for analysis of variance indicated significant results, the Unpaired *t*-test with Welch’s correction was used to analyze significant differences, and the Welch-corrected *t*-test parameters were indicated. For the assessment of weight, we also applied a paired *t*-test for repeated measures. Analyses were performed using Prism, version 5 (GraphPad Software Inc., San Diego, CA, USA). Linear regression analysis and Pearson’s correlation coefficient were computed to assess the potential relationship between 2 different variables.

## 3. Results

### 3.1. Body Weight and Blood Glucose Levels in ZDF and Lean Rats

At the time of arrival at 7 weeks of age, ZDF rats presented a significantly increased body weight when compared to lean control rats (*p* < 0.0001; t = 5.660; df = 14; F(7,7) = 3.825) (Table 1). At the end of the experiments (at 32 weeks of age), this situation was inverted (*p* = 0.0088; t = 3.081 df = 13; F(7,6) = 1.308). Indeed, to analyze the progression along time of the increase in the animals’ weight, the Paired t-test for repeated measures was applied and indicates a significant difference for both lean (*p* < 0.0001; t = 30.32; df = 7) and ZDF rats (*p* < 0.0001; t = 19.23; df = 6). ZDF rats presented a significant increase in glycemia at the end of experiments (*p* = 0.0001; Welch-corrected t = 8.575 df = 6; F(6,7) = 141.3) (Table 1).

### 3.2. Memory Assessment and Glucocorticoid Evaluation

**Figure 2:**
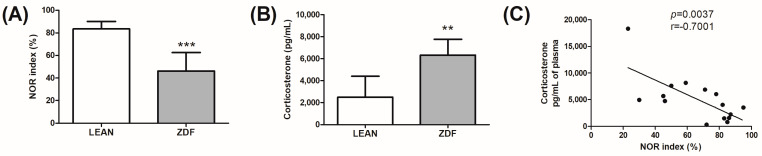
Memory deficit of diabetic animals detected with the novel object recognition (NOR) test and corticosterone plasma levels. (**A**): NOR index; (**B**): corticosterone plasma levels; and (**C**): correlation analysis between NOR index and corticosterone levels. Analysis was performed in non-diabetic controls (lean) and Zucker diabetic fatty rats (ZDF) at 8 months of age. The columns represent the mean ± SD (n = 8–6). Normal distribution of data was evaluated with Kolmogorov–Smirnov test; then, two-tailed Student’s *t*-test analysis was applied. If the F test for variance was significant, an unpaired t-test with Welch’s correction was used: ** *p* < 0.01 and *** *p* < 0.001 vs. lean.

To study memory dysfunction, we evaluated lean control animals and ZDF rats at 31 weeks of age using the novel object recognition (NOR) test. ZDF rats showed a significant reduction in the NOR index when compared with lean controls (*p* = 0.0008; Welch-corrected t = 5.632 df = 7; F(6,7) = 5.853) (Figure 2A). We also assessed plasma corticosterone levels in lean and ZDF rats. As expected, corticosterone levels were increased in ZDF rats as compared with controls (*p* = 0.0014; t = 4.124 df = 12; F(7,5) = 1.790) (Figure 2B) and significantly correlated with memory impairment (*p* = 0.0037; r(13) = −0.7001; F(1,13) = 12.50) (Figure 2C).

### 3.3. Neuroactive Steroid Levels in ZDF and Lean Rats

Neuroactive steroid levels were analyzed in plasma and the hippocampus using liquid chromatography–tandem mass spectrometry (LC–MS/MS) (Table 2). In plasma, we observed increased levels of the first steroid PREG (*p* = 0.0054; Welch-corrected t = 4.243 df = 6; F(6,6) = 15.83), of PROG (*p* = 0.0012; Welch-corrected t = 5.772 df = 6; F(6,6) = 13.53), and its metabolite DHP (*p* = 0.006; Welch-corrected t = 4.158 df = 6; F(6,5) = 18,13). Moreover, in agreement with the hypogonadism associated with T2DM, we detected decreased levels of T (*p* = 0.0029; Welch-corrected t = 3.907 df = 10; F(7,5) = 4.53). In the brain tissue, we observed a similar increase in PREG (*p* = 0.0166; t = 2.822 df = 11; F(5,6) = 1.039) and PROG levels (*p* = 0.0186; Welch-corrected t = 3.434 df = 5; F(5,5) = 20.51) and a reduction in T levels (*p* = 0.0012; t = 4.349 df = 11; F(5,6) = 1.466). Indeed, we observed decreased levels of ALLO (*p* = 0.0096; Welch-corrected t = 3.529 df = 7; F(6,4) = 15.02) and increased levels of DHEA (*p* = 0.0318; Welch-corrected t = 3.237 df = 4; F(4,5) = 32.64) in the hippocampus of ZDF rats as compared with the lean controls.

### 3.4. Correlation Analysis among Neuroactive Steroid Levels and NOR Index in ZDF and Lean Rats

**Figure 3:**
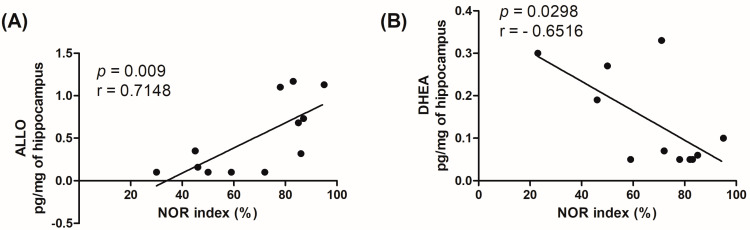
Correlation analysis. The levels of allopregnanolone (ALLO—(**A**)) and dehydroepiandrosterone (DHEA—(**B**)) in the hippocampus are in correlation with the NOR index. The *p*-value (*p*) and Pearson’s r (r) are reported.

To evaluate the contribution of brain-specific alterations of neuroactive steroid levels on the memory deficits observed in our model, we performed a correlation analysis (Figure 3). We plotted the levels of hippocampal ALLO or DHEA versus the values of the NOR index. Correlation analyses showed that both ALLO (*p* = 0.009; r(10) = 0.511; F(1,10) = 10.45) (Figure 3A) and DHEA (*p* = 0.0298; r(9) = −0.6516; F(1,9) = 6.64) (Figure 3B) levels are correlated with the NOR index, albeit in opposite directions. Considering the *p*-value, ALLO levels correlated with higher significance to the levels of DHEA.

### 3.5. Hippocampal Alterations Induced by T2DM

**Figure 4:**
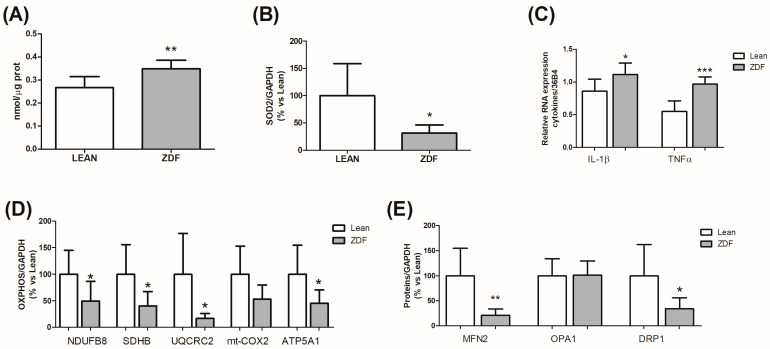
Alterations in the hippocampus. Thiobarbituric acid reactive substances (TBARS) (**A**), protein expression levels of superoxide dismutase 2 (SOD2) (**B**), gene expression levels of the pro-inflammatory cytokines interleukin 1 beta (IL−1β) and tumor necrosis factor-alpha (TNFα) (**C**), protein expression levels of the mitochondrial oxidative phosphorylation system (OXPHOS) and, in particular, of the proteins NDUFA8B, SDHB, UQCRC2, mt-COX2, and ATP5A1 (**D**) and of proteins involved in mitochondrial dynamics, such as mitofusin−2 (MFN2), mitochondrial dynamin-like GTPase (OPA1), and dynamin-related protein (DRP1), (**E**) were analyzed in the hippocampus of non-diabetic controls (lean) or of Zucker diabetic fatty rats (ZDF) at 8 months of age. The columns represent the mean ± SD (n = 8–6). Normal distribution of data was evaluated with Kolmogorov–Smirnov test; then, two-tailed Student’s *t*-test analysis was applied. If the F test for variance was significant, an unpaired *t*-test with Welch’s correction was used: * *p* < 0.05, ** *p* < 0.01, and *** *p* < 0.001 vs. lean.

To investigate the molecular alterations that may be associated with memory deficit, we first explored the presence of oxidative stress in the hippocampal region of lean control animals and ZDF rats. Indeed, our previous experience in experimental models of hyperglycemia indicates that those parameters are interesting targets in diabetic encephalopathy [21,22]. As reported in Figure 4, the TBARS assay indicates a statistically significant increase (*p* = 0.0081; t = 3.296 df = 10; F(5,5) = 1.60) in the oxidative stress response in the hippocampus of ZDF rats as compared to the lean control group (Figure 4A). In agreement, the protein levels of superoxide dismutase type 2 (SOD2) were significantly decreased (*p* = 0.024; Welch-corrected t = 3.001 df = 6; F(6,6) = 16.04) (Figure 4B). The increase in oxidative stress may be related to the increased inflammatory signature. Thus, we detected the gene expression of the proinflammatory cytokines interleukin 1 beta (IL−1β; *p* = 0.0218; t = 2.633 df = 12; F(6,6) = 1.062) and tumor necrosis factor-alpha (TNFα; *p* = 0.0001; t = 5.560 df = 12; F(6,6) = 2.042), which were both increased in the hippocampus of ZDF rats as compared to the control animals (Figure 4C). To further investigate the presence of oxidative stress, we also evaluated possible mitochondrial dysfunction. Thus, mitochondrial factors involved in ATP production (e.g., proteins involved in the mitochondrial respiratory chain complexes), and mitochondrial dynamics were evaluated using Western blotting. The expression of proteins involved in complexes I (*p* = 0.0486; t = 2.218 df = 11; F(5,6) = 1.464), II (*p* = 0.0234; t = 2.568 df = 13; F(7,6) = 4.285), III (*p* = 0.0188; Welch-corrected t = 3.040; df = 7; F(7,5) = 64.87), and V (*p* = 0.0314; t = 2.411 df = 13; F(7,6) = 4.761) was significantly reduced (Figure 4D); moreover, we reported an impairment in mitochondrial dynamics, since the expression of MFN2 (*p* = 0.0056; Welch-corrected t = 3.941 df = 7; F(7,5) = 18.94) and DRP1 (*p* = 0.0349; Welch-corrected t = 2.611 df = 7; F(6,5) = 8.168) were both significantly decreased (Figure 4E), while those of OPA1 were not affected.

### 3.6. Diversity and Composition of Gut Microbiota Are Affected by T2DM

**Figure 5:**
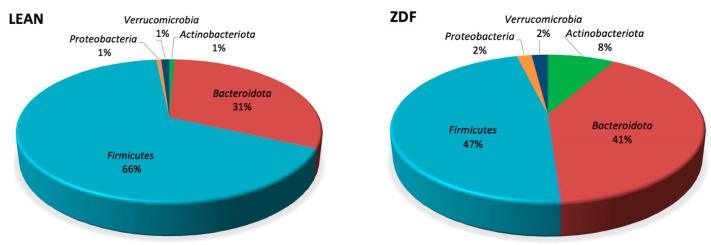
A pie chart of *phyla* in stool samples in lean and ZDF groups.

**Figure 6:**
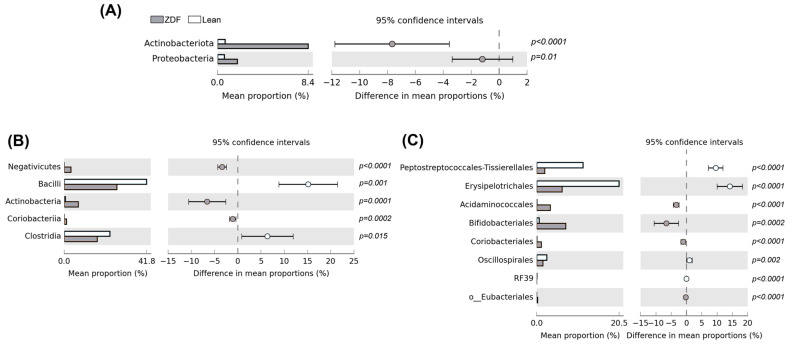
Mean proportions and differences in mean proportion are shown for significantly different bacterial species for *phylum* (**A**), *class* (**B**), and *order* (**C**) taxonomy ranks in lean and ZDF rats. The black bars highlight the 95% confidence intervals of each analysis.

The composition of gut microbiota was characterized using 16S rRNA next-generation sequencing analysis. To explore changes in the intestinal microbiota due to T2DM, we first analyzed α- and β-diversity in stools collected 24 h before sacrifice. We observed a statistically significant decrease in α-diversity in ZDF, as assessed by the Shannon index (Benjamini–Hochberg-adjusted *p*-value = 0.037), Faith’s phylogenetic distance (Benjamini–Hochberg-adjusted *p*-value = 0.008) and observed OTUs (Benjamini–Hochberg-adjusted *p*-value = 0.003). The evenness index showed a similar trend but failed to reach statistical significance (Benjamini–Hochberg-adjusted *p*-value = 0.132). In addition, β-diversity analysis revealed statistically significant differences based on both unweighted (Benjamini–Hochberg-adjusted *p*-value = 0.001) and weighted (Benjamini–Hochberg-adjusted *p*-value = 0.001) UniFrac distance, as well as Bray–Curtis dissimilarity (Benjamini–Hochberg-adjusted *p*-value = 0.001). In the predominant *phyla* observed in stool samples (i.e., *Firmicutes* lean: 66.30 ± 8.07; ZDF: 47.20 ± 3.78 and *Bacteroidota* lean: 31.19 ± 8.35; ZDF: 40.52 ± 3.69), no significant differences were observed in the two groups (Figure 4). However, as suggested by the pie chart, bacterial taxa belonging to *Actinobacteriota* and *Proteobacteria* were significantly increased in ZDF animals (Figure 5 and Figure 6A).

Indeed, T2DM affects several bacterial *classes,* such as *Negativicutes*, *Bacilli,* and *Clostridia* as well as *classes* belonging to *Actinobacteria* and *Coriobacteriia*, which were grouped in the *phylum Actinobacteriota* (Figure 6B). Specifically, in these *classes*, we distinguished the *order* of *Bifidobacteriales* in the former and *Coriobacteriales* in the latter (Figure 6C). Moreover, at the order level, *Acidaminococcales* and *Eubacteriales* were also increased by T2DM (Figure 6C). In contrast, the trend of *Peptostreptococcales-Tissierellales*, *Erysipelotrichales*, *Oscillospirales*, and *RF39* was decreased in ZDF rats when compared with controls (Figure 6C). 

**Figure 7:**
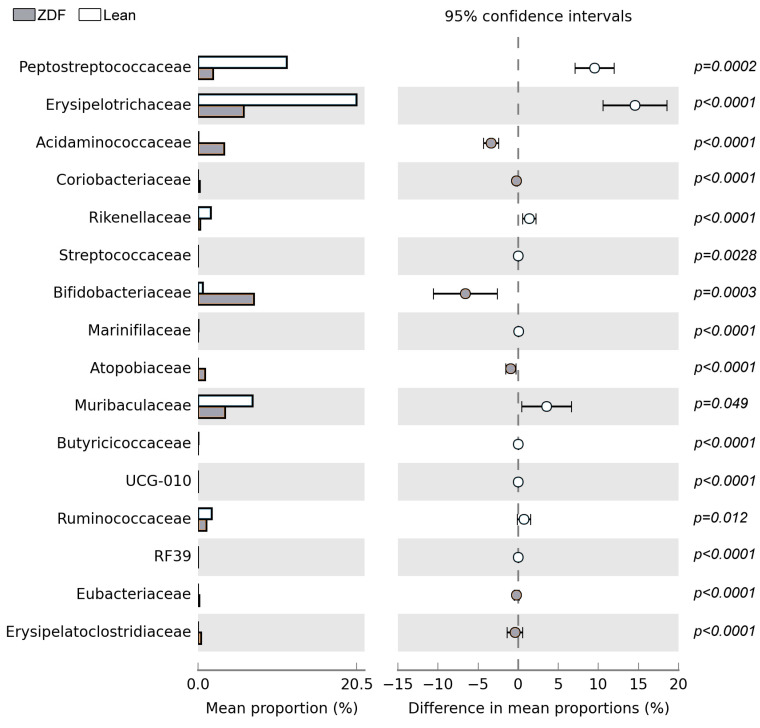
Mean proportions and differences in mean proportion are shown for significantly different bacterial species for *family* taxonomy ranks in lean and ZDF rats. The black bars highlight the 95% confidence intervals of each analysis.

*Bifidobacteriales* and *Coriobacteriales* at the *order* level, and *Bifidobacteriaceae* and *Coriobacteriaceae* at the *family* level, were significantly increased in ZDF rats when compared to controls (Figure 6C and Figure 7). Additionally, among the families that are significantly altered by the diabetic conditions, we detected that *Coriobacteriaceae* and *Erysipelatoclostridiaceae* were increased in ZDF rats, while *Muribaculaceae*, *UCG−010*, *Ruminococcaceae,* and *RF39* were decreased (Figure 7). Furthermore, 34 *genera* were affected in the ZDF rats. 

**Figure 8:**
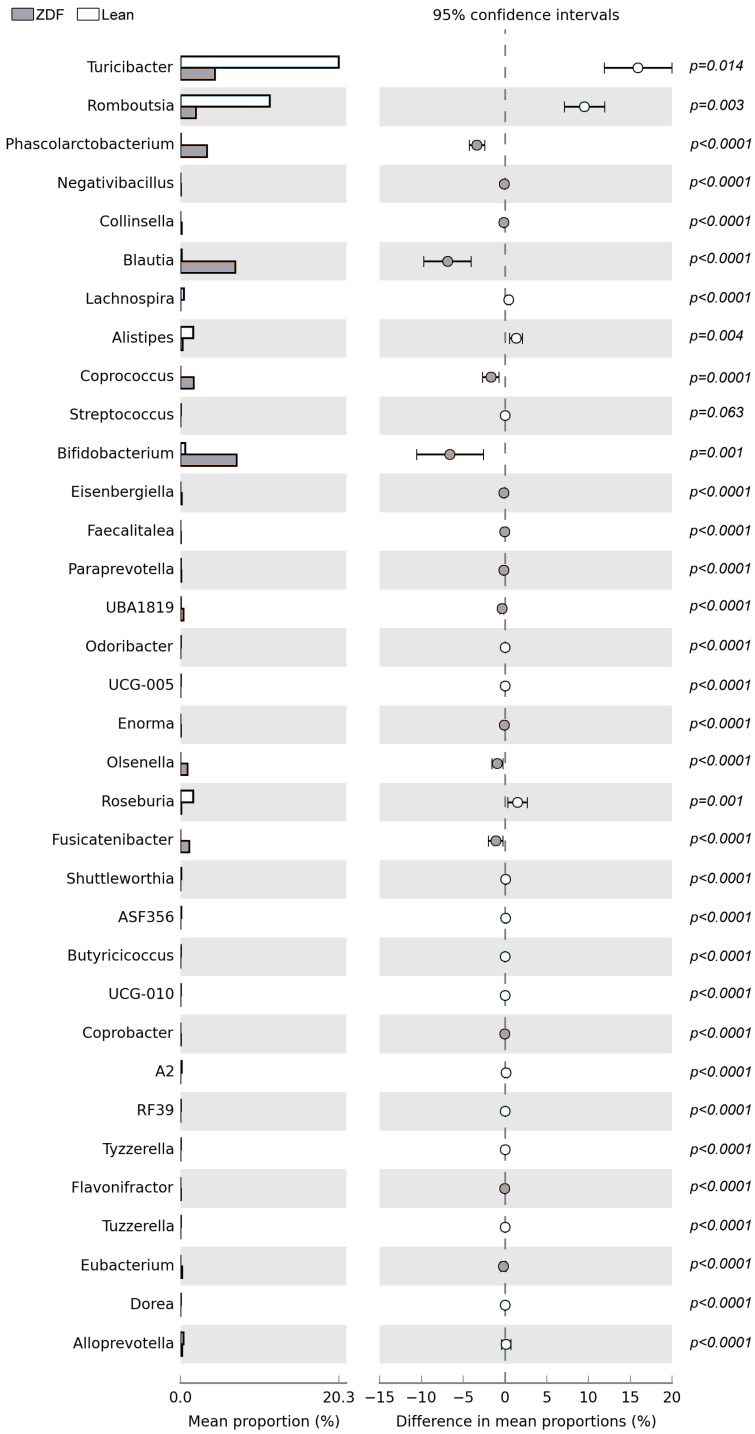
Mean proportions and differences in mean proportion are shown for significantly different bacterial species for *genus* taxonomy ranks in lean and ZDF rats. The black bars highlight the 95% confidence intervals of each analysis.

In particular, *Turicibacter* (i.e., *Erysipelotrichaceae*), *Romboutsia* (i.e., *Peptostreptococcaceae*), *Alistipes* (i.e., *Rikenellaceae*), *Streptococcus* (i.e., *Streptococcaceae*), *Odoribacter* (i.e., *Marinifilaceae*), *Butyricicoccus* (i.e., *Butyricoccaceae*) as well as *Lachnospira*, *Roseburia*, *Shuttleworthia*, *Tyzzerella*, *A2*, *UCG−005*, *ASF356*, *Dorea*, *UCG−010*, *RF39*, *Tuzzerella*, and *Alloprevotella* were significantly decreased in T2DM when compared with controls (Figure 6 and Figure 7). On the other hand, *Phascolarctobacterium* (i.e., *Acidaminocaccaceae*), *Bifidobacterium* (i.e., *Bifidobacteriaceae*), *Olsenella* (i.e., *Atopobiaceae*), *Eubacterium* (i.e., *Eubacteriaceae*) as well as *Negativibacillus*, *UBA1819*, *Collinsella*, *Enorma*, *Blautia*, *Fusicatenibacter*, *Eisenbergiella*, *Coprococcus*, *Faecalitalea*, *Paraprevotella*, *Coprobacter*, and *Flavonifractor* were significantly increased in T2DM when compared to controls (Figure 7 and Figure 8).

### 3.7. Correlation between Plasma Corticosterone and Hippocampal Allopregnanolone Levels in Genera Affected by T2DM and Correlated to NOR Index

We then performed a correlation analysis between steroid levels and microbiota (genera both affected by T2DM and significantly correlated with the NOR index) to bring out the taxa simultaneously related to corticosterone in the plasma and ALLO in the hippocampus. Interestingly, among 34 genera altered in the ZDF group (Figure 8), only 22 showed a significant correlation with the NOR index (Table 3). 

Out of these 22 *genera*, Pearson’s r revealed a significant linear correlation between the frequency of *Collinsella*, *Paraprevotella*, *Phascolarctobacterium*, *Turicibacter,* and *Romboutsia* and steroid concentration (i.e., corticosterone levels in plasma and ALLO levels in the hippocampus) (Table 4). 

## 4. Discussion

The male ZDF rat is a commonly used model for T2DM because of the fa/fa genotype, leading to leptin receptor deficiency. This produces obesity and hyperglycemia early in life; moreover, animals display insulin resistance and hyperglycemia due to reduced activity of insulin signaling and age-dependent degeneration in pancreatic β-cell function [54,55]. This model shows signs of peripheral diabetic complications [56], together with defects in hippocampal plasticity and impaired learning and memory [57]. In agreement, here we show that ZDF rats at 8 months of age present memory impairment when assessed for the NOR test. This is in line with previous findings [58], where younger animals displayed a decrease in the recognition index when compared to lean controls.

The pathogenesis of diabetes-associated cognitive decline is related to several factors, such as brain insulin resistance, mitochondrial damage, oxidative stress, calcium homeostasis imbalance, and inflammatory response [59,60]. In addition, another primary mechanism includes increased levels of glucocorticoids, which are corticosterone in rodents and cortisol in humans. Thus, their levels are increased in diabetic patients [61] as well as in animal models of DM, like the ZDF rats [62]. In agreement, here we showed that the levels of corticosterone are increased also in our experimental model; moreover, here we detected a negative correlation between these levels and memory function. Of note, a correlation between cortisol levels and decreased hippocampal volume in T2DM patients has been reported [63]. Moreover, Stranahan and colleagues [64] demonstrated that in an insulin-resistant model, elevated levels of corticosterone impaired neurogenesis, synaptic plasticity, and learning. Therefore, when normal levels of glucocorticoids were maintained, the changes in hippocampal plasticity and function were improved, indicating that cognitive impairment in this model is a possible result of glucocorticoid-mediated deficits in brain functions. Chronic exposure to glucocorticoids can have an inflammatory outcome in the CNS, promoting proinflammatory cell migration and cytokine production [65]. Accordingly, in an insulin-resistant model, corticosterone induced increased microglia activation with subsequent accumulation of the cytokines IL−1β and TNFα within the hippocampus [66]. Overall, these data suggest a role for glucocorticoids in diabetes-associated cognitive decline.

Besides glucocorticoids, it is well known that memory is also controlled by other neuroactive steroids, as extensively reviewed elsewhere [29]. Additionally, it has been reported already in other experimental models of diabetes that the levels of these molecules are altered [67], and this in turn produces alterations in the brain’s functionality, specifically in the hippocampus and the cerebral cortex [21,22,30]. To our knowledge, no indications about neuroactive steroid levels in the brain and their relation to memory function from experimental models in T2DM have been reported so far. Interestingly, we showed that neuroactive steroid levels are altered in the hippocampus of ZDF rats and that some alterations are specific to this brain region and not related to plasma levels. This is the case for ALLO and DHEA, where their levels are decreased and increased by diabetes, respectively. Conversely, the levels of PREG, PROG, and T are similarly regulated in the brain and the plasma, suggesting that the levels in the hippocampus may be partially due to uptake or lack of it, in the case of T, from the plasma. On this basis, we moved to analyze some molecular aspects that may be altered in the hippocampus by diabetes. Indeed, we found increased levels of oxidative stress measured by the TBARS assay and decreased levels of the antioxidant protein SOD2. In line with these results, an increase in ROS formation and a decrease in antioxidant defense have been detected in ZDF brains at younger ages (17 weeks) [68]. Moreover, others reported that pathway-specific gene expression profiling in the same experimental model showed significant increases in oxidative stress and inflammation [69]. Indeed, in our model, neuroinflammation (e.g., increased gene expression levels of the proinflammatory cytokines IL−1β and TNFα) was detected in the ZDF hippocampus, in agreement with what was reported by others in the same experimental model [70]. To further investigate the presence of oxidative stress, and considering the decreased expression of SOD2 which is required to control oxidative stress in mitochondria, we evaluated possible mitochondrial dysfunction. We reported decreased expression of the proteins related to complexes I, II, III, and V as well as of MFN2 and DRP1, overall suggesting mitochondrial dysfunction. Similar dysfunctions in the diabetic brain have also been reported by others. For example, in the ZDF rat brain, increased oxidative stress and reduced mitochondrial respiratory functions were observed [71]. A reduction in the OXPHOS complexes has been observed also by Jolivalt and colleagues [58] in the ZDF cerebral cortex. Moreover, a role for hippocampal mitochondrial impairment in the cognitive deficit observed in ZDF rats has been recently suggested [72]. Interestingly, ALLO levels may be involved in all these processes. As already published, ALLO is a neuroactive steroid with antioxidant potential and which directly regulates SOD2 levels [73,74]. Moreover, ALLO exerts anti-inflammatory functions [26] and has a well-known protective action on mitochondria [75,76]. The importance of ALLO in the context of DM is further confirmed by the results here obtained. Indeed, as indicated by correlation analysis, this steroid, compared to DHEA, showed a higher correlation value with the NOR index. Of note, DHEA levels present a negative correlation with the NOR index, suggesting a negative action on diabetic encephalopathy. This is quite surprising, since the protective and antioxidant actions of this steroid are well known [77], even if its actions have been better described during aging [78,79]. Moreover, in this paper, we reported increased hippocampal levels of PREG and PROG that, as stated above, show a similar pattern in plasma. Also in this case, it is interesting to note that both steroids are considered neuroprotective factors, with antioxidant and anti-inflammatory properties [27,80,81,82]. Thus, the observed increase can be seen as an attempt to cope with the damage in the brain region considered; however, it is not sufficient to promote the necessary protection against oxidative stress, neuroinflammation, and mitochondrial damage. This implies that an easier therapeutic approach could be addressed by directly increasing the levels of this PROG metabolite, which are reduced in our model. In addition, decreasing the levels of the precursor of androgens (i.e., DHEA) may be detrimental due to the low levels of T, here reported, and the possibility of worsening the hypoandrogenism/hypogonadism already reported in T2DM [83]. 

On the other hand, a strict interaction among brain functions, including memory, DM, and microbiota, is also well ascertained. Indeed, changes in microbiota composition in T2DM patients [84,85] and in ZDF rats have been already reported [86,87,88,89,90], and it has been proposed that these changes contribute to insulin resistance and metabolic syndrome-developing progress. However, whether they are related to memory alterations and/or to the steroid environment in T2DM, has not been yet reported. Thus, to fill this gap of knowledge, we also explored microbiota alterations in ZDF rats to identify possible candidates, such as *genera* modified by this pathological condition and related to memory dysfunction and steroid changes. This concept agrees with the recent hypothesis that steroids are possible players in the communication between the CNS and gut microbiota [91,92]. Indeed, and as reported above, the lack of microbiota can influence neuroactive steroid levels, as observed in germ-free mice [32], and gonadectomy and sex affect microbiota composition [93]. However, it remains to be determined if the changes in the neuroactive steroid levels are the cause or rather the consequence of dysbiosis. Additionally, a mutual relationship between intestinal bacteria and glucocorticoids has been reported for T2DM [94].

Thus, here we reported higher levels of the *genus Collinsella* that were also increased in T2DM patients [95] with symptomatic atherosclerosis and hyperinsulinemia [96,97]. Interestingly, a negative correlation between this pro-inflammatory *genus* and the NOR index was highlighted in ZDF rats. Moreover, a positive and negative linear correlation with corticosterone and allopregnanolone, respectively, was reported. 

Similarly, *Paraprevotella* belonging to the *Bacteroidota phylum* can promote inflammatory disease features [98]. Even if a causal and potential disease-triggering mechanism is still unclear, interestingly here we reported a positive correlation with corticosterone (Table 4). Moreover, the abundance of this aging-associated *genus* [99] is related to dysbiosis and T2DM [100]. In agreement, here we reported that a rise in this *taxon* was negatively correlated with the NOR index and ALLO levels (Table 4). 

Furthermore, a rise in *Phascolarctobacterium* belonging to the *Negativicutes* class was also observed. Like *Paraprevotella* and *Collinsella*, this bacterium is positively correlated with corticosterone levels and negatively correlated with both the NOR index and ALLO levels (Table 3 and Table 4). Of note, *Negativicutes* can convert succinate to propionate [101,102]; although possibly helpful for maintaining human health, current evidence suggests that an excess of this short-chain fatty acid may play a role in AD [103].

Finally, a lower relative abundance of *Romboutsia* and *Turicibacter* was observed in ZDF rats as in the streptozotocin-induced diabetic rat model [104] with consolidated cognitive dysfunction, indicating a possible pathological link.

Interestingly, the *genera* discussed here can be considered relevant in our model and to be further explored, based on how they behave in relation to steroid levels and the NOR index. Indeed, all the *genera* with a negative correlation with the NOR index showed a similar negative correlation with ALLO levels and a positive correlation with corticosterone levels (i.e., *Collinsella*, *Paraprevotella,* and *Phascolarctobacterium*). In contrast, those *genera* that are positively correlated with the NOR index are similarly correlated to ALLO levels and negatively with glucocorticoid levels (i.e., *Romboutsia* and *Turicibacter*). Thus, even if future investigations are necessary to confirm the causal association between these microbes and steroid metabolites, this study highlights a possible direction for the treatment of diabetic encephalopathy through modification of the gut microbiota and host steroid environment.

## 5. Conclusions

In conclusion, the data obtained here for the ZDF model of T2DM show that diabetic encephalopathy, demonstrated by memory deficit and alteration in molecular parameters in the hippocampus, is present along with increased plasma glucocorticoid levels. But besides this result, which agrees with previous observations [62], we reported here for the first time that neuroactive steroid levels and microbiota composition are altered in this experimental model. Interestingly, the decreased levels of ALLO, the alterations in *Collinsella*, *Paraprevotella*, *Phascolarctobacterium*, *Romboutsia,* and *Turicibacter*, and the increase in corticosterone and memory impairment present a mutual correlation. These results may suggest new scenarios in the management of diabetes cognitive impairment. Limitations of the study are represented by the lack of a direct demonstration of a causal role of steroid level impairment and microbiota changes in diabetic encephalopathy. Therefore, future experiments will investigate if treatment with the neuroactive steroid ALLO and/or supplementation with specific probiotics may exert a possible protective effect on diabetic encephalopathy.

## Figures and Tables

**Table 1:** Rat weight at the beginning (7 weeks of age), at the end of the experiment (32 weeks of age), and glycemia at the sacrifice of non-diabetic controls (lean) and of Zucker diabetic fatty rats (ZDF).

	Lean	ZDF
Weight (g)—7 weeks	169.3 ± 17.2	207.9 ± 8.8 ***
Weight (g)—32 weeks	426.7 ± 32.4	377.4 ± 28.2 **
Glycemia (mg/dL)—32 weeks	114.5 ± 10.6	524.8 ± 126.2 ***

Data (n = 8–7) are expressed as mean ± SD. Normal distribution of data was evaluated with Kolmogorov–Smirnov test; then, two-tailed Student’s *t*-test analysis was applied. If the F test for variance was significant, an unpaired t-test with Welch’s correction was used: ** *p* < 0.01 and *** *p* < 0.001 vs. lean.

**Table 2:** Neuroactive steroid levels assessed in the plasma and hippocampus of non-diabetic controls (lean) and of Zucker diabetic fatty (ZDF) rats at 8 months of age.

	Plasma		Hippocampus	
	Lean	ZDF	Lean	ZDF
PREG	0.34 ± 0.27	2.1 ± 1.07 **	4.39 ± 1.80	7.23 ± 1.83 *
PROG	1.16 ± 0.78	7.62 ± 2.85 **	1.04 ± 0.48	4.14 ± 2.16 *
DHP	0.33 ± 0.13	1.19 ± 0.53 **	0.86 ± 0.63	1.55 ± 0.62
ALLO	0.37 ± 0.30	0.4 ± 0.15	0.75 ± 0.42	0.16 ± 0.11 **
ISOALLO	0.12 ± 0.06	0.14 ± 0.06	0.37 ± 0.27	<0.1
DHEA	<0.05	<0.05	0.06 ± 0.02	0.23 ± 0.11 *
T	1.62 ± 0.72	0.5 ± 0.34 **	0.99 ± 0.19	0.48 ± 0.23 **
DHT	0.06 ± 0.02	<0.05	0.37 ± 0.30	0.27 ± 0.18
3α-DIOL	0.07 ± 0.03	0.06 ± 0.02	0.36 ± 0.34	0.44 ± 0.15
17β-E	0.03 ± 0.02	<0.02	0.05 ± 0.02	0.03 ± 0.02

Data (n = 8–5) are expressed as pg/μL ± SD in the case of plasma (left) and pg/mg ± SD in the case of the hippocampus (right). Detection limits were 0.02 pg/μL or pg/mg for testosterone (T) and 17β-estradiol (17β-E), 0.05 pg/μL or pg/mg for pregnenolone (PREG), progesterone (PROG), 3α-diol, dehydroepiandrosterone (DHEA), dihydrotestosterone (DHT); 0.1 pg/μL or pg/mg for allopregnanolone (ALLO) and isoallopregnanolone (ISOALLO); 0.25 pg/μL or pg/mg for dihydroprogesterone (DHP). Normal distribution of data was evaluated with Kolmogorov–Smirnov test; then, two-tailed Student’s *t*-test analysis was applied. If the F test for variance was significant, an unpaired *t*-test with Welch’s correction was used: * *p* < 0.05 and ** *p* < 0.01 vs. lean.

**Table 3:** List of *genera* relative abundance affected by T2DM and significantly correlated with the NOR index.

Genus	NOR Index
*Turicibacter*	r(13) = 0.883; *p* = <0.000; F(1,13) = 45.98
*UBA1819*	r(13) = −0.832; *p* = 0.000; F(1,13) = 28.19
*Negativibacillus*	r(13) = −0.818; *p* = 0.000; F(1,13) = 26.2
*Collinsella*	r(13) = −0.816; *p* = 0.000; F(1,13) = 25.96
*Bifidobacterium*	r(13) = −0.813; *p* = 0.000; F(1,13) = 25.39
*Romboutsia*	r(13) = 0.798; *p* = 0.000; F(1,13) = 22.72
*Paraprevotella*	r(13) = −0.768; *p* = 0.001; F(1,13) = 18.71
*Blautia*	r(13) = −0.707; *p* = 0.003; F(1,13) = 13.03
*Faecalitalea*	r(13) = −0.703; *p* = 0.004; F(1,13) = 12.69
*Phascolarctobacterium*	r(13) = −0.700; *p* = 0.004; F(1,13) = 12.46
*Flavonifractor*	r(13) = −0.691; *p* = 0.004; F(1,13) = 11.9
*Alistipes*	r(13) = 0.664; *p* = 0.007; F(1,13) = 10.23
*Enorma*	r(13) = −0.662; *p* = 0.007; F(1,13) = 10.12
*Eisenbergiella*	r(13) = −0.631; *p* = 0.012; F(1,13) = 8.62
*Lachnospira*	r(13) = 0.610; *p* = 0.016; F(1,13) = 7.71
*Coprococcus*	r(13) = −0.578; *p* = 0.024; F(1,13) = 6.51
*Streptococcus*	r(13) = 0.577; *p* = 0.024; F(1,13) = 6.50
*Odoribacter*	r(13) = 0.572; *p* = 0.026; F(1,13) = 6.31
*Eubacterium*	r(13) = −0.567; *p* = 0.028; F(1,13) = 6.16
*UCG-010*	r(13) = 0.548; *p* = 0.035; F(1,13) = 5.58
*Fusicatenibacter*	r(13) = −0.536; *p* = 0.039; F(1,13) = 5.25
*RF39*	r(13) = 0.517; *p* = 0.049; F(1,13) = 4.74

Data are reported as Pearson’s r(df) with *p*-value and F(df1, df2). The genera are reported in descending order where the highest is the most correlated with the NOR index.

**Table 4:** Correlation of corticosterone and allopregnanolone levels with *genera* relative abundance.

Genus	Corticosterone	Allopregnanolone
*Collinsella* (+)	r(12) = 0.633; *p* = 0.015; F(1,12) = 8.026	r(10) = −0.586; *p* = 0.045; F(1,10) = 5.226
*Paraprevotella* (+)	r(12) = 0.667; *p* = 0.009; F(1,12) = 9.633	r(10) = −0.620 *p* = 0.031; F(1,10) = 6.258
*Phascolarctobacterium* (+)	r(12) = 0.723; *p* = 0.004; F(1,12) = 13.15	r(10) = −0.708; *p* = 0.010; F(1,10) = 10.04
*Turicibacter* (−)	r(12) = −0.687; *p* = 0.007; F(1,12) = 10.72	r(10) = 0.831; *p* = 0.001; F(1,10) = 22.22
*Romboutsia* (−)	r(12) = −0.544; *p* = 0.045; F(1,12) = 5.032	r(10) = 0.816; *p* = 0.001; F(1,10) = 19.9

Values in the table report Pearson’s r(df) with *p*-value and F(df1, df2). The symbols (+) and (−) indicate increased and decreased, respectively in ZDF vs. lean.

## Data Availability

The data presented in this study are available on request from the corresponding author.
